# Supplementary material for: Amplification of future energy demand growth due to climate change
Source: Nat Commun. 2019 Jun 24;10:2762. doi: 10.1038/s41467-019-10399-3 (PMC6591298; doi:10.1038/s41467-019-10399-3)
Supplement: Supplementary file 1 — Supplementary Information [file 41467_2019_10399_MOESM1_ESM.pdf]

Supplementary Information for

Amplification of Future Energy Demand Growth due to Climate Change

van Ruijven, et al., Nature Communications, 2019

Supplementary Note 1: Sector Definitions

Our definition of economic sectors matches the definition used by the International Energy Agency, and it reflects the final consumption of energy by final sectoral users.

- Agriculture includes the sectors ISIC 01–03. Agriculture/forestry includes deliveries to users classified as agriculture, hunting and forestry by the ISIC, and energy consumed by such users whether for traction (excluding agricultural highway use), power or heating (agricultural and domestic).
- The commercial sector includes the sectors ISIC 33; 36–39; 45–47; 53; 55; 56; 58–66; 68–75; 77–82; 84 (excl. 8422); 85–88; 90–96; 99.
- Industry includes the sectors ISIC 241, 2431: Iron and steel; 20–21: Chemical and petrochemicals excl. petrochemical feedstocks; 242, 2432: Non-ferrous metal basic industries; 23: Non-metallic minerals; 29–30: Transport equipment; 25–28: Machinery, fabricated metal products, machinery and equipment other than transport equipment; 07, 08, 099: Mining (excl. fuels) and quarrying; 10–12: food and tobacco; 17–18: Paper, pulp and print; 16: Wood and wood products (other than pulp and paper); 41–43: Construction; 13–15: Textile and leather; 22, 31–32: Manufacturing n.e.c.
- The residential sector includes the sectors ISIC 97–98. Heat pumps operated within the residential sector where heat is not sold are not considered a transformation process and are included here Transportation ISIC 49–51: Consumption in transport covers all transport activity (in mobile engines) regardless of the economic sector to which it contributes.

Supplementary Note 2: Income grouping of countries

Fig. 5 and Fig. S5 use income grouping of countries into the World Bank income classes of low income countries, lower middle income countries, upper middle income countries and high income countries. The most recent World Bank definitions of these groups are in year 2014 Atlas method USD GDP per capita levels of:

- <$1045 low income
- <$4125 lower middle income
- <$12735 upper middle income

The GDP projections for the SSPs (from the OECD) are provided in year 2005 USD, also using the Atlas method. However, converting these cutoff levels from year 2014 to year 2005 dollars is not straightforward given the definition of the Atlas method. An estimated adjustment based on which countries are around the cutoff levels of each category lead to the following definition in year 2005 dollars:

- <$2000 low income
- <$6000 lower middle income
- <$15000 upper middle income

In Fig. 5, we use lower middle income countries as proxy for high challenges to adaptation, upper middle income countries as proxy for moderate challenges to adaptation and high income countries as proxy for low challenges to adaptation.

Supplementary Note 3: Reconciling Bottom-Up and Top-Down Estimates of the Impact of Climate Change on Electricity Demand

The error-correction model employed by De Cian and Sue Wing (2019) - hereafter DCSW - is a reparameterization of the lagged dependent variable specification

$$q_{s,i,t} = \alpha_{s,i} + \Sigma_z \begin{Bmatrix} \lambda_{s,z}\left(q_{s,i,t-1} * D_{z(i)}\right) + \beta_{s,Y,z}\left(y_{i,t} * D_{z(i)}\right) + \\ \beta_{s,L,z}\left(L_{i,t} * D_{z(i)}\right) + \beta_{s,H,z}\left(H_{i,t} * D_{z(i)}\right) \end{Bmatrix} + \varepsilon_{s,i,t} \qquad \text{(S1)}$$

where s, i, z and t index sectors, countries, climatic zones and years, respectively, and D denotes a dummy variable that assigns countries to a temperate or tropical climate zone, y and q denote the logarithm of per capita GDP and sectoral consumption of the type of fuel under consideration, and L and H denote the annual sum of days with population-weighted low temperatures (daily average < 12.5 °C) and high temperatures (daily average > 27.5 °C) over each year. The estimated parameter α_i is a country fixed effect, and $\boldsymbol{\beta}_Y$, $\boldsymbol{\beta}_L$ and $\boldsymbol{\beta}_H$ are the coefficients of interest. Our projections are based on the estimates in Table 2, which are the long-run responses $\beta_{s,Y,z}/(1-\lambda_{s,z})$, $\beta_{s,L,z}/(1-\lambda_{s,z})$ and $\beta_{s,H,z}/(1-\lambda_{s,z})$.

For days with mild weather (12.5 °C < T < 27.5 °C), C and H are both zero and $q_{s,i} = \alpha_{s,i} + \beta_{s,Y,z}(y_{i,t} * D_{z(i)})$ meaning that a country's demand remains at its conditional mean per capita level determined by idiosyncratic factors and income. (DCSW also include a vector of fuel prices as controls but these elasticities were estimated with precision, and were frequently dropped from the regressions because of gaps in the relevant series. Where $\boldsymbol{\beta}_L$ and $\boldsymbol{\beta}_H$ are identified, the

result is a piecewise nonlinear spline that traces out a piecewise-linear, generally U-shaped, response (cf DCSW, Fig. 1).

Recent climate econometric studies of electric power demand use large samples of observed load and temperature at fine spatial- and temporal scales to estimate coefficients on multiple bins of temperature that trace out a nonlinear demand response. Auffhammer et al (2017) [46] - hereafter ABH - show that the latter can be well approximated by linear schedules for increasing high temperatures and decreasing low temperatures, outside of an intermediate moderate temperature zone. This point is apparent from visual inspection of ABH Fig. 1 (or as well in Wenz et al, 2017: Figs. 2 and 3).

We exploit this insight to approximate the empirical model underlying ABH Fig. 1 using the local degree-day specification

$$V_{\ell,d} = \gamma_\ell + \delta_{L,\ell} \max\left(12.5 - T_{\ell,d}, 0\right) + \delta_{C,\ell} \max\left(15 - T_{\ell,d}, 0\right)$$
$$+\delta_{W,\ell} \max(T_{\ell,d} - 18,0) + \delta_{H,\ell} \max(T_{\ell,d} - 21,0) + \vartheta_{\ell,d} \qquad \text{(S2)}$$

where now $\ell(i)$ indexes fine geographic scale locations within the USA, $d(t)$ indexes the days within each year, V and γ denote the location's observed and conditional mean hourly energy demand, the subscripts C and W indicate "cool" (as opposed to cold) days with intermediate low temperatures (daily average 12.5 °C < T < 16 °C) and "warm" (as opposed to hot) days with intermediate high temperatures (daily average 21 °C < T < 27.5 °C). The parameters of interest

are the local load ramps with temperature for cold, cool, warm and hot days, $\boldsymbol{\delta}_L$, $\boldsymbol{\delta}_C$, $\boldsymbol{\delta}_W$ and $\boldsymbol{\delta}_H$, which can be backed out from the coordinates of points on the average load-temperature responses in the figure (see Supplementary Table 6).

We note that, due to the insurmountable limitations of IEA data, DCSW do not find exposures in the ranges 12.5 °C < T < 15 °C, 15 °C < T < 17.5 °C, 20 °C < T < 22.5 °C, 22.5 °C < T < 25 °C or 25 °C < T < 27.5 °C (or the collapsed ranges 12.5 °C < T < 17.5 °C, 20 °C < T < 27.5 °C) to have statistically significant effects. Notwithstanding this, the local and geographically averaged demand responses can be reconciled. ABH do not distinguish short- and long-run effects, accordingly, for comparability we utilize the static responses reported in DCSW Table 11. Let N denote local population. If the latter parameters absorb the effects on demand of moderate as well as extreme temperatures, then for cold and hot days

$$\bar{\beta}_L = \mathbb{E}\left\{\frac{\partial \log[\Sigma_\ell \Sigma_d\big(\gamma_\ell + \delta_{L,\ell}\max\big(12.5 - T_{\ell,d}, 0\big) + \delta_{C,\ell}\max\big(15 - T_{\ell,d}, 0\big)\big)/\Sigma_\ell N_{\ell,t}]}{\partial\left[\Sigma_\ell\left(N_{\ell,t} * \Sigma_d\big(T_{\ell,d(t)} < 12.5\big)\right)/\Sigma_\ell N_{\ell,t}\right]}\right\} \tag{S3a}$$

$$\bar{\beta}_H = \mathbb{E}\left\{\frac{\partial \log[\Sigma_\ell \Sigma_d\big(\gamma_\ell + \delta_{W,\ell}\max(T_{\ell,d} - 18,0) + \delta_{H,\ell}\max(T_{\ell,d} - 21,0)\big)/\Sigma_\ell N_{\ell,t}]}{\partial\left[\Sigma_\ell\left(N_{\ell,t} * \Sigma_d\big(T_{\ell,d(t)} > 27.5\big)\right)/\Sigma_\ell N_{\ell,t}\right]}\right\} \tag{S3b}$$

Whereas if geographically averaged demand responses capture only the effects of temperature extremes, they underestimate the weather responsiveness of demand

$$\underline{\beta}_L = \mathbb{E}\left\{\frac{\partial \log[\Sigma_\ell \Sigma_d(\gamma_\ell + \delta_{L,\ell} \max(12.5 - T_{\ell,d}, 0))/\Sigma_\ell N_{\ell,t}]}{\partial \left[\Sigma_\ell \left(N_{\ell,t} * \Sigma_d\left(T_{\ell,d(t)} < 12.5\right)\right)/\Sigma_\ell N_{\ell,t}\right]}\right\} \quad \text{(S4a)}$$

$$\underline{\beta}_H = \mathbb{E}\left\{\frac{\partial \log[\Sigma_\ell \Sigma_d(\gamma_\ell + \delta_{H,\ell} \max(T_{\ell,d} - 27.5,0))/\Sigma_\ell N_{\ell,t}]}{\partial \left[\Sigma_\ell \left(N_{\ell,t} * \Sigma_d\left(T_{\ell,d(t)} > 27.5\right)\right)/\Sigma_\ell N_{\ell,t}\right]}\right\} \quad \text{(S4b)}$$

Eqs. (S3) and (S4) elucidate that local weather-insensitive energy consumption, the sub-national distribution of population and the distribution average daily temperatures above and below DCSW's hot and cold cutoffs potentially drive a wedge between the local and geographically averaged estimates.

To operationalize eqs. (S3) and (S4), we collect data for the period 2005-2018 on annual county populations from the US Bureau of Economic Analysis, as well as gridded historical hourly 2m air temperature from the North American Land Data Assimilation System (NLDAS) forcing file A, which are mapped to 487 counties in PJM and 194 counties in ERCOT. Weather-insensitive demand corresponding to the omitted 15 °C < T < 18 interval, γ, is not observed. We approximate it by the hourly systemwide demand for days with average temperature 15°C < T < 18°C over the period 2006-2014 covered by ABH's dataset: 30,036 MW for ERCOT and 67,022 MW for PJM. Because of the prevalence of moderate temperature days, the omitted interval in ABH's response is associated with a large fraction of demand, leaving weather-sensitive demand to account for just 17.5% and 11.8% of ERCOT and PJM total annual total, respectively.

Supplementary Table 7 illustrates how DCSW's elasticities compare with (S3) and (S4). To maximize comparability, we aggregate DCSW's temperature responses for the residential, commercial and industrial sectors. At least in the US context, the reconciled temperature semi-elasticities built up from heterogeneous "bottom-up" estimates for PJM and ERCOT are of the same overall magnitude as their counterpart "top-down" semi-elasticities identified from variation across temperate countries. However, the concern is that DCSW's weighted average response to cold days is insignificant, while the response to hot days exceeds the estimates implied by ABH.

The key question is the magnitude of bias this divergence introduces into projections of the impact of warming on energy demand. From ABH's levels specification (S2), given vectors of daily temperatures at multiple locations under current and future climates, $\bar{T}_{\ell,d}$ and $\bar{T}'_{\ell,d}$, the bottom-up impact is given by the fractional increase in annual electricity demand

$$I_\ell = \frac{8760 * \gamma_\ell + \Sigma_d 24 * \begin{pmatrix} \delta_{L,\ell} \max\left(12.5 - \bar{T}'_{\ell,d}, 0\right) + \delta_{C,\ell} \max\left(15 - \bar{T}'_{\ell,d}, 0\right) + \\ \delta_{W,\ell} \max\left(\bar{T}'_{\ell,d} - 18,0\right) + \delta_{H,\ell} \max\left(\bar{T}'_{\ell,d} - 21,0\right) \end{pmatrix}}{8760 * \gamma_\ell + \Sigma_d 24 * \begin{pmatrix} \delta_{L,\ell} \max\left(12.5 - \bar{T}_{\ell,d}, 0\right) + \delta_{C,\ell} \max\left(15 - \bar{T}_{\ell,d}, 0\right) + \\ \delta_{W,\ell} \max\left(\bar{T}_{\ell,d} - 18,0\right) + \delta_{H,\ell} \max\left(\bar{T}_{\ell,d} - 21,0\right) \end{pmatrix}} \qquad \text{(S5)}$$

Note how in both the numerator and denominator of (S5), total annual demand is partitioned into weather-insensitive and weather-responsive components, respectively. To undertake an apples-to-apples comparison of the impacts of ABH's total demand in levels against DCSW's sectoral

demand in logarithms, the two sets of projections should be constructed with similar conditional means. DCSW's conditional mean per capita load for the entire United States diverges from that for ERCOT and PJM. To reconcile the two sets of estimates following the partitioning strategy in (S5), two pieces of ancillary information are necessary to transform sectoral semi-elasticities into demand in levels: each sector's fraction of demand, σ_s (Supplementary Table 7, column 4), and the weather-responsive fraction of total demand, ω. The result is the comparable impact metric is

$$\begin{aligned}
\tilde{I}_\ell &= \frac{8760 * \gamma_\ell * \langle 1 + \frac{\omega_\ell}{1-\omega_\ell}\left[\Sigma_s \sigma_s * \exp\left\{\beta_{s,L}\Sigma_d\left(1*\left(\bar{T}'_{\ell,d} < 12.5\right)\right) + \beta_{s,H}\Sigma_d\left(1*\left(\bar{T}'_{\ell,d} > 27.5\right)\right)\right\}\right]\rangle}{8760 * \gamma_\ell * \langle 1 + \frac{\omega_\ell}{1-\omega_\ell}\left[\Sigma_s \sigma_s * \exp\left\{\beta_{s,L}\Sigma_d\left(1*\left(\bar{T}_{\ell,d} < 12.5\right)\right) + \beta_{s,H}\Sigma_d\left(1*\left(\bar{T}_{\ell,d} > 27.5\right)\right)\right\}\right]\rangle} \\
&= \frac{(1-\omega_\ell) + \omega_\ell\left[\Sigma_s \sigma_s * \exp\left\{\beta_{s,L}\Sigma_d\left(1*\left(\bar{T}'_{\ell,d} < 12.5\right)\right) + \beta_{s,H}\Sigma_d\left(1*\left(\bar{T}'_{\ell,d} > 27.5\right)\right)\right\}\right]}{(1-\omega_\ell) + \omega_\ell\left[\Sigma_s \sigma_s * \exp\left\{\beta_{s,L}\Sigma_d\left(1*\left(\bar{T}_{\ell,d} < 12.5\right)\right) + \beta_{s,H}\Sigma_d\left(1*\left(\bar{T}_{\ell,d} > 27.5\right)\right)\right\}\right]}
\end{aligned} \tag{S6}$$

For counties in the ERCOT and PJM territories, we extract daily average temperatures from NASA NEX GDDP dataset's downscaled and bias corrected 0.25° gridded maximum and minimum daily temperatures simulated by CMIP5 runs of the CCSM4 climate model. To calculate (S5) and (S6), we compare average daily temperatures for the late-century 2090-99 ($\bar{T}'_{\ell,d}$) to ABH's 2006-15 study period ($\bar{T}_{\ell,d}$).

The results, shown in Supplementary Figure 10, are reasonable, with eq. (S5) projecting increases in total energy demand of 11.2% for ERCOT and 7.7% for PJM, in excellent agreement with ABH: Table 1. By comparison, aggregated impacts based on DCSW's estimates

that are significant at the 15% level were found to be larger for ERCOT and smaller for PJM, and to exhibit greater variance. The scatterplots indicate an upward bias relative to ABH's transformed elasticities in hot regions (ERCOT), consistent with Table S8. The pattern of significance of DCSW's estimates implies that as locations' temperature distributions shift rightward, their electricity demand responds more elastically to the positive effect of increases hot days, but responds less elastically or completely inelastically to the negative impact of reductions in cold days.

These findings are encouraging, suggesting that, even at fine spatial scales, DCSW's semi-elasticities form a credible basis for projecting the impacts of climate change on energy demand. Although such projections require ancillary data to pin down local conditional means, and even then do not precisely replicate the empirical responses and patterns of impacts generated by more sophisticated high temporal frequency/fine spatial scale econometric models, the fact that a simple, global model estimated on data that are incommensurate and far coarser is able to match these projections' broad patterns attests to the validity of our approach.

Still, our analysis highlights the caveat that the local conditional mean energy consumption and the fraction of weather-sensitive demand are crucial parameters that determine the magnitude of impact. However, these are not observed for the various fuel × sector combinations in grid cells across the world. Our projection methodology is based on the assumption that in the long run $\omega \rightarrow 1$, so that, employing the long-run elasticities in Table 1, eq. (S6) collapses to the sectoral impact metric

$$\tilde{I}_{s,\ell} = exp\left\{\begin{matrix}\beta_{s,L}/(1-\lambda_s) * \left(\Sigma_d \left(1 * \left(\bar{T}'_{\ell,d} < 12.5\right)\right) - \Sigma_d\left(1 * (\bar{T}' < 12.5)\right)\right) + \\ \beta_{s,H}/(1-\lambda_s) * \left(\Sigma_d \left(1 * \left(\bar{T}'_{\ell,d} > 27.5\right)\right) - \Sigma_d \left(1 * \left(\bar{T}_{\ell,d} > 27.5\right)\right)\right)\end{matrix}\right\} \quad \text{(S7)}$$

which we use to quantify the fractional increase in 2050 energy demand due to climate change. This expression likely overestimates the response of demand to warming, but there is a pervasive lack of information on which to assess the magnitude and geographic distribution of the biases to which it may be subject. For this reason, it is prudent to interpret the impacts in the text as worst-case projections especially in tropical and subtropical regions, conditional on the extent of warming.

Supplementary Figure 1

Number of people exposed to changes in energy demand by sector, energy carrier and total for the mean and all 21 CMIP5 models by 2050 under RCP8.5 and SSP5

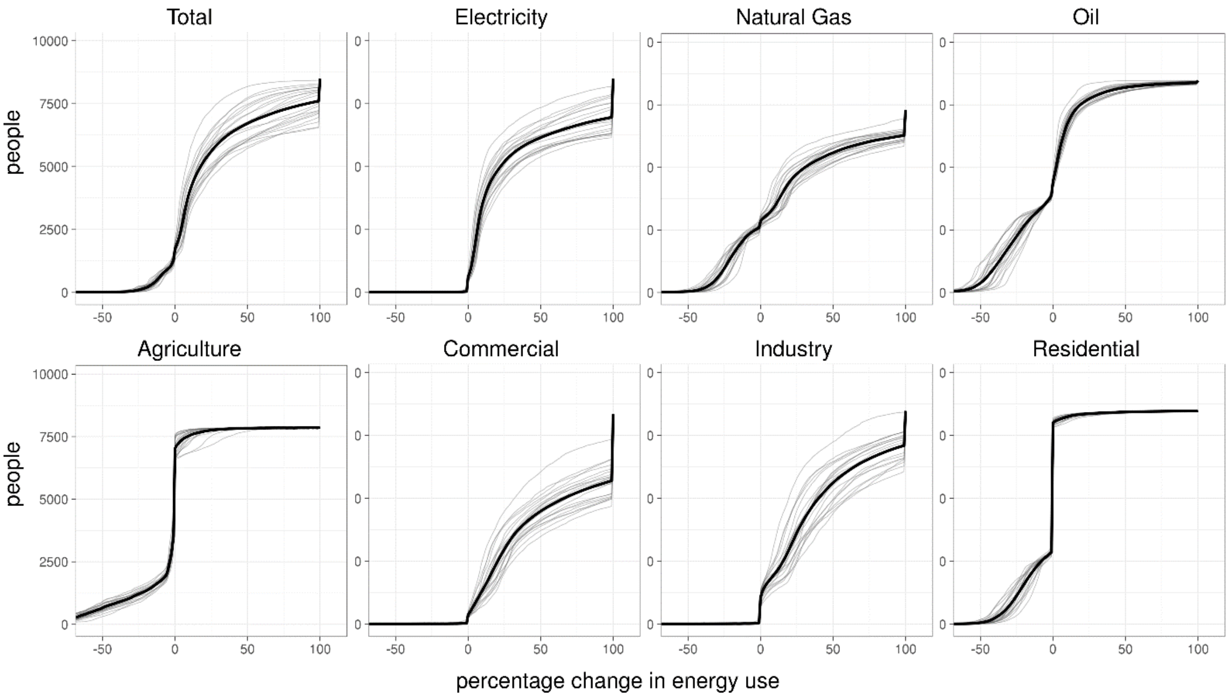

Supplementary Figure 2

Number of people exposed to changes in energy demand by sector, energy carrier and total for the mean and all 21 CMIP5 models by 2050 under RCP4.5 and SSP5

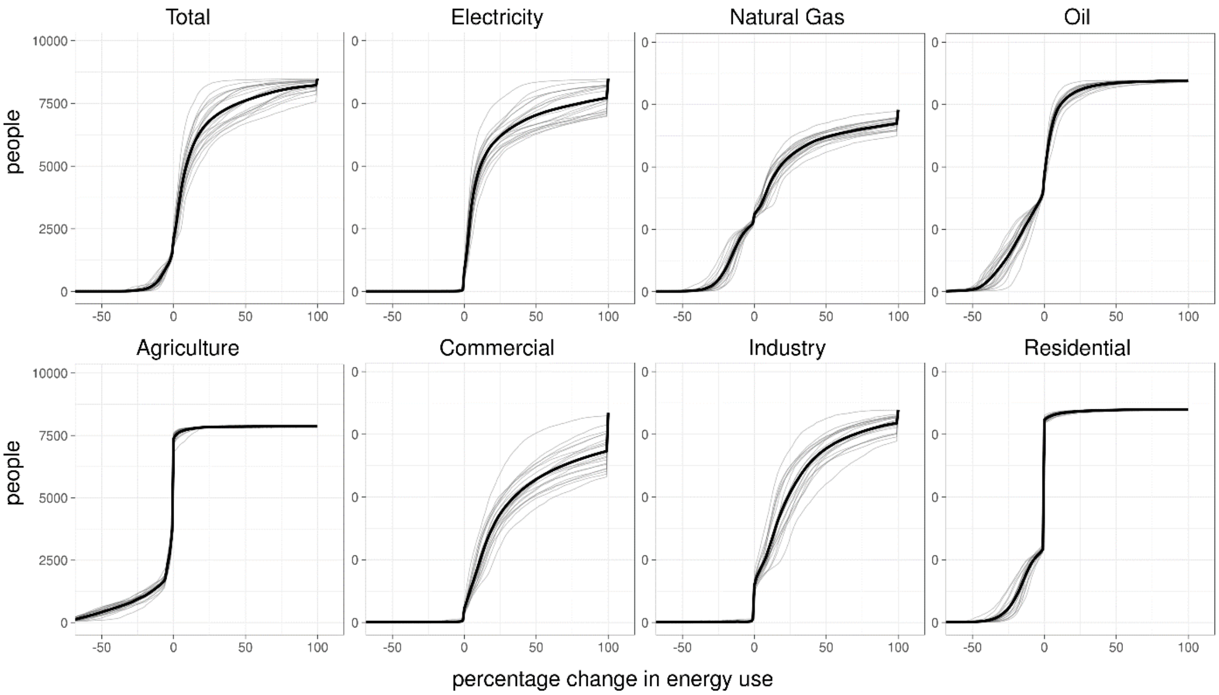

Supplementary Figure 3

Cumulative distribution of the number of people exposed to percentage change in climate-related final energy demand by country GDP per capita. Lines indicate the multi-model mean (thick lines) and all individual 21 CMIP5 models (thin lines) by 2050 under RCP4.5. Present day World Bank definitions for GDP per capita were used to classify countries in income categories, which we linked to adaptation challenges.

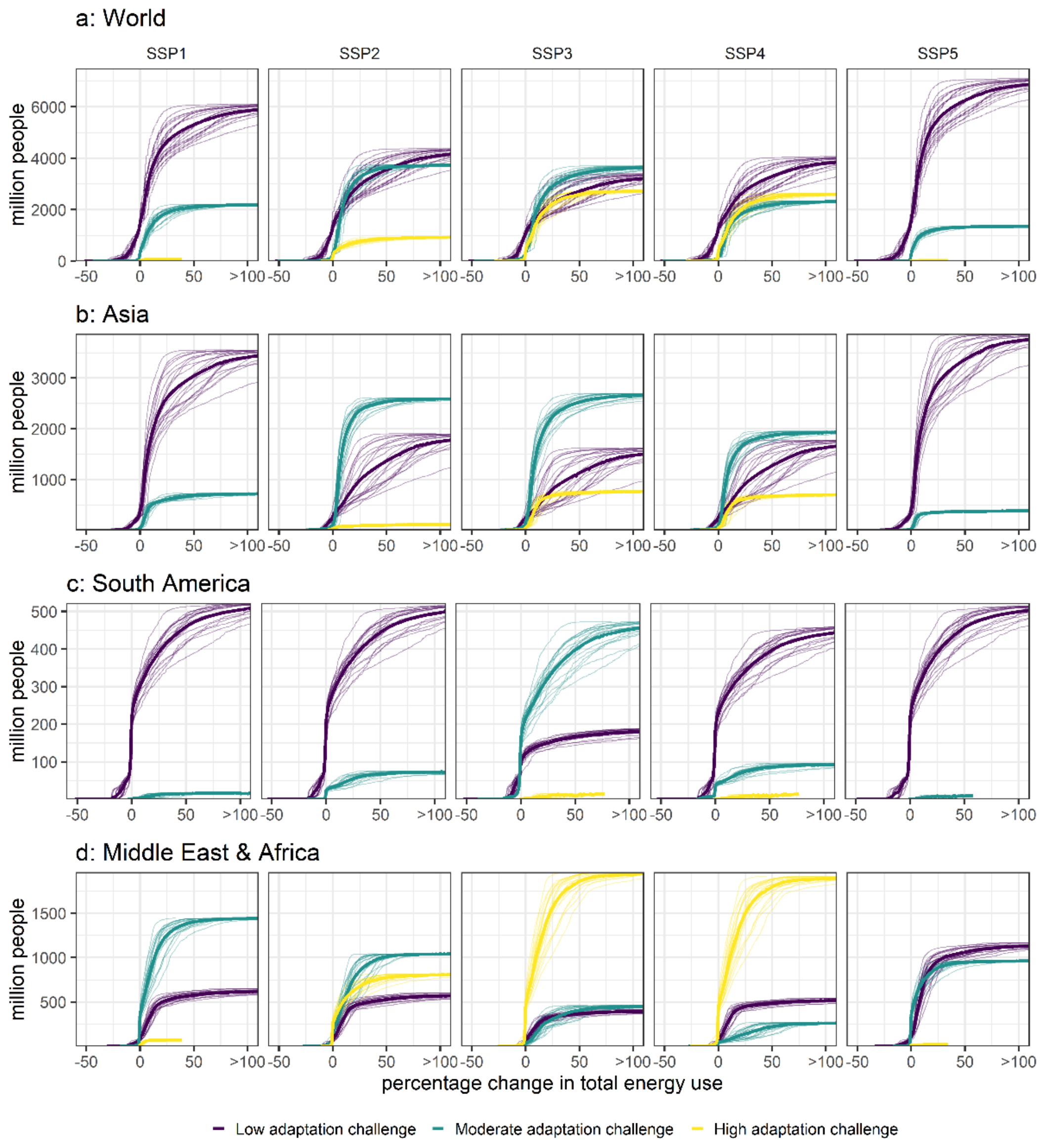

Supplementary Figure 4

Comparison between base-year data used in our analysis (red dots) and in six Integrated Assessment Model (box and whisker) realizations of final energy demand by sector (residential and services (together in buildings), industry) and energy carriers (electricity, natural gas and petroleum products) by 2010 for the World. The bottom panels indicate that our study has a different definition for liquids than the IAMs.

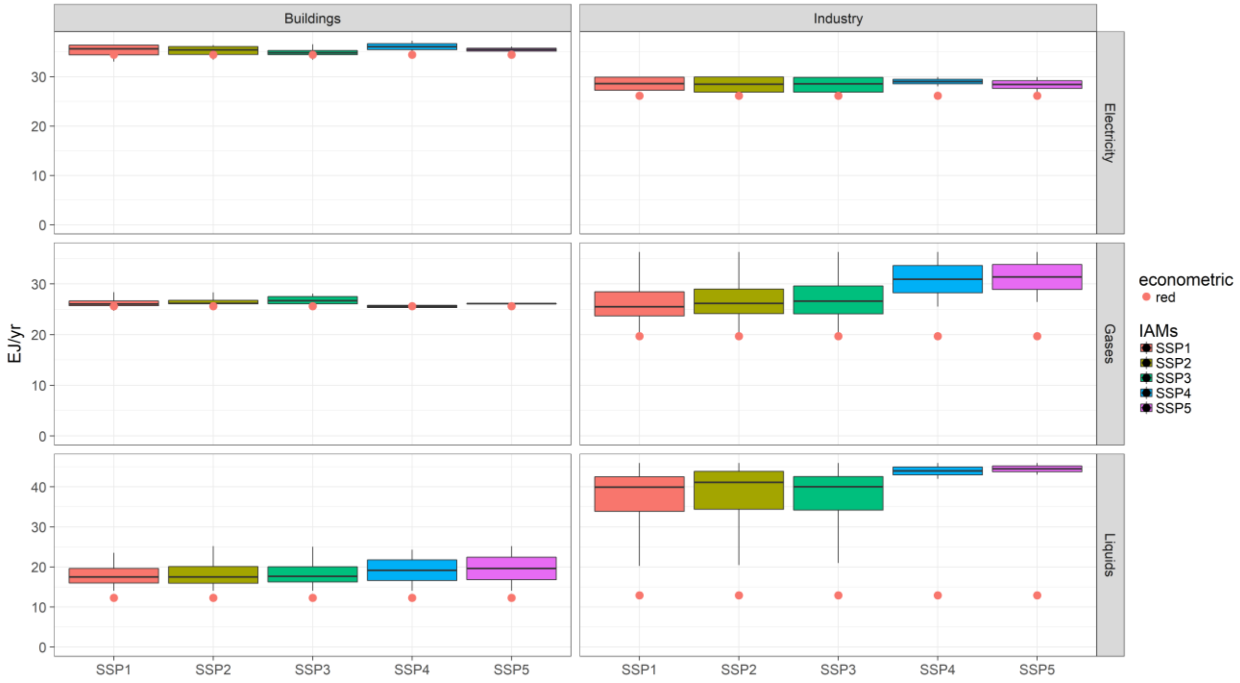

Supplementary Figure 5

Comparison between econometric model projections (red dots) and in six Integrated Assessment Model (box and whisker) realizations of final energy demand by sector (residential and services (together in buildings) and industry) and energy carrier (electricity, natural gas and petroleum products) by 2050 for the World under five SSPs.

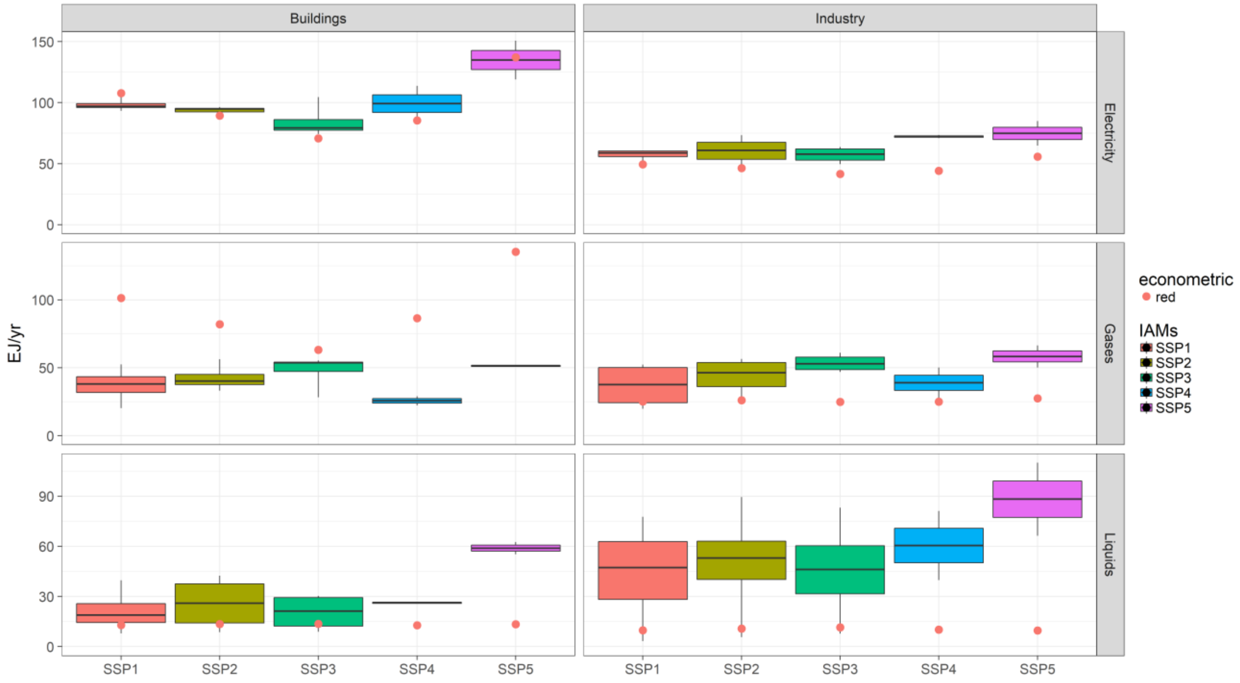

Supplementary Figure 6

Changes in the days with mean temperatures above 27.5°C and below 12.5°C under RCP4.5 for 21 ESMs and the mean. Contrary to RCP8.5, which is shown in the main text, this lower climate change scenarios leads to a smaller increase in the number of hot days and a minor decrease in the number of cold days.

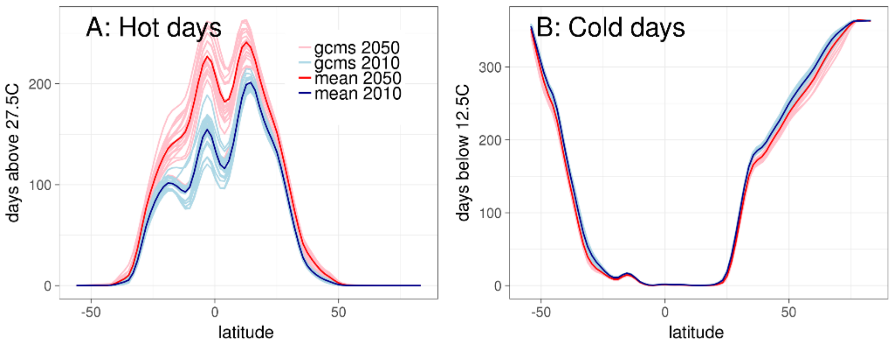

Supplementary Figure 7

Total energy demand exposed to changes in cold and hot days under RCP4.5 (bottom) and RCP8.5 (top). In the detailed panels (A, B, E, F) the blue lines depict present-day energy demand, and the red lines depict SSP5 baseline energy demand, brown lines indicate energy use under SSP5 after impacts of climate change (mean and all 21 ESMs) exposed to certain changes in hot and cold days. Aggregate panels (C, D, G, H) show the multi-ESM mean for all five SSPs. Impacts from climate change are shown for combined changes in hot and cold days.

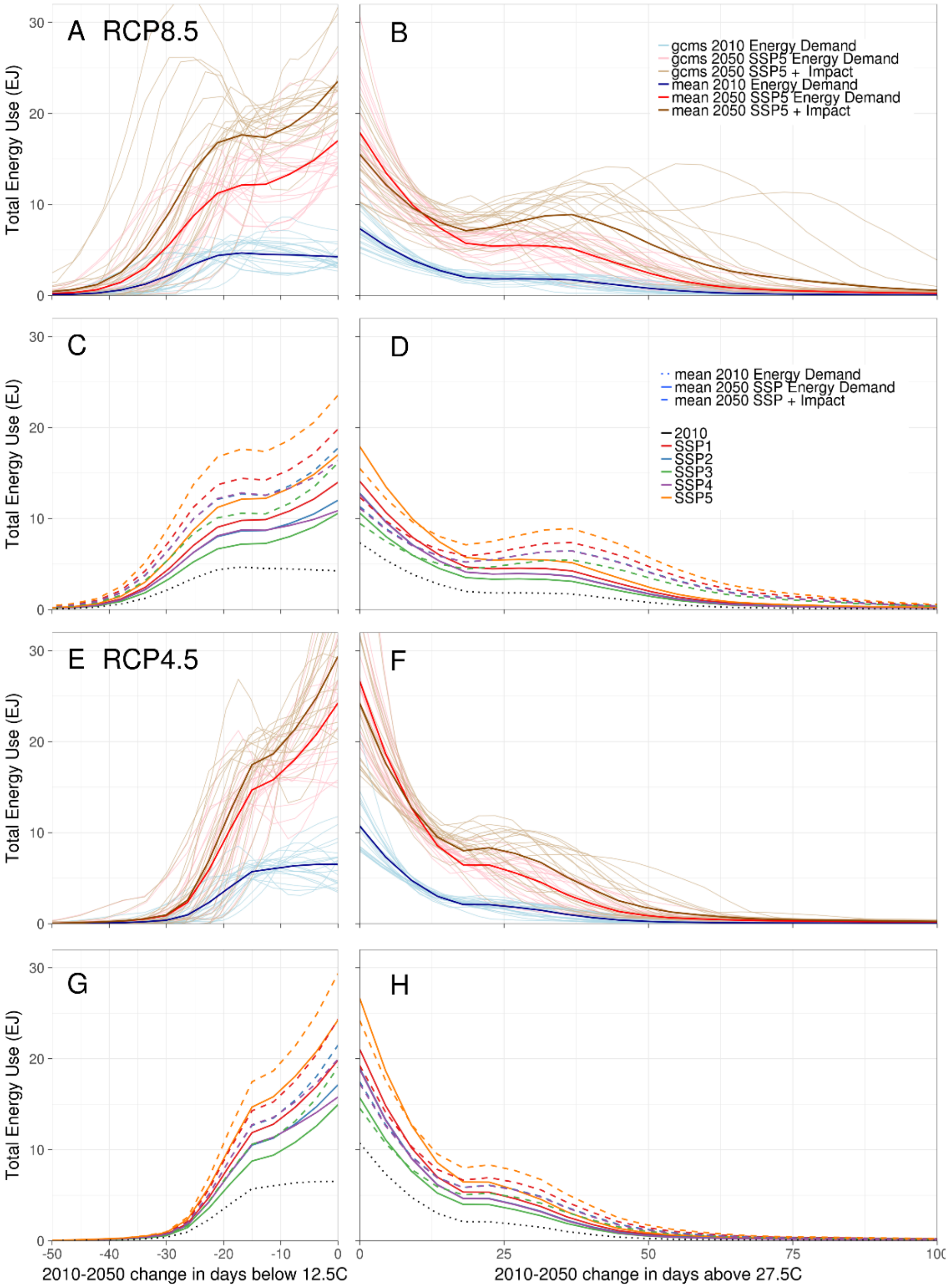
A RCP8.5
B
C
D
E RCP4.5
F
G
H
Total Energy Use (EJ)
gcms 2010 Energy Demand
gcms 2050 SSP5 Energy Demand
gcms 2050 SSP5 + Impact
mean 2010 Energy Demand
mean 2050 SSP5 Energy Demand
mean 2050 SSP5 + Impact
mean 2010 Energy Demand
mean 2050 SSP Energy Demand
mean 2050 SSP + Impact
2010
SSP1
SSP2
SSP3
SSP4
SSP5
2010-2050 change in days below 12.5C
2010-2050 change in days above 27.5C

251

252

Supplementary Figure 8

Fuel *x* sector contributions to global total energy demand amplification due to climate change around 2050, under RCP4.5 and across SSPs. Solid bars represent the median of 21 ESM model simulations, error bars represent the interquartile range of change in energy demand across 21 ESM simulations.

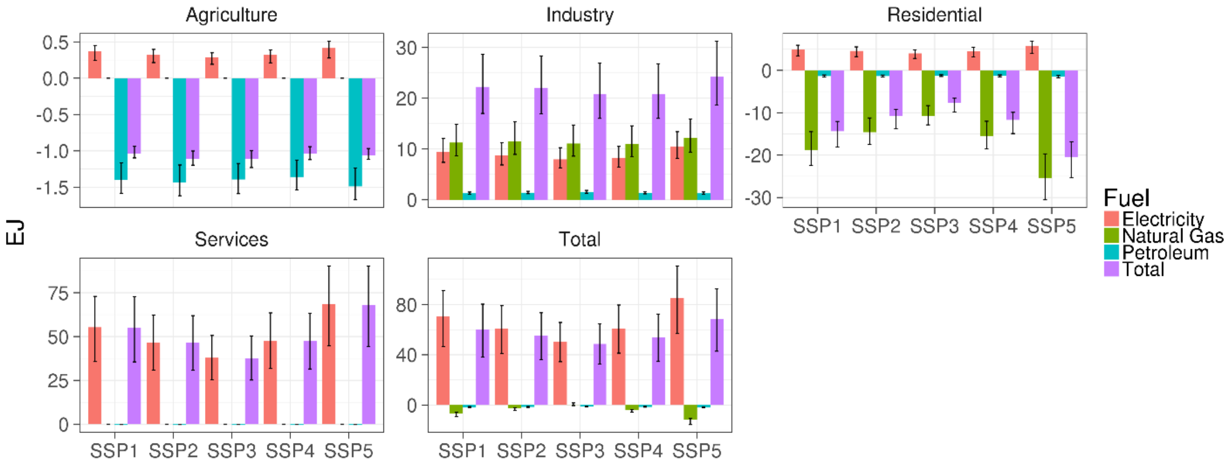

Supplementary Figure 9

Number of climate models that agree on total climate-related energy demand to increase or decrease by more than 0, 10, 25 or 50% by 2050 under RCP4.5 and SSP5 as result of the 21 CMIP5 model ensemble of temperature projections

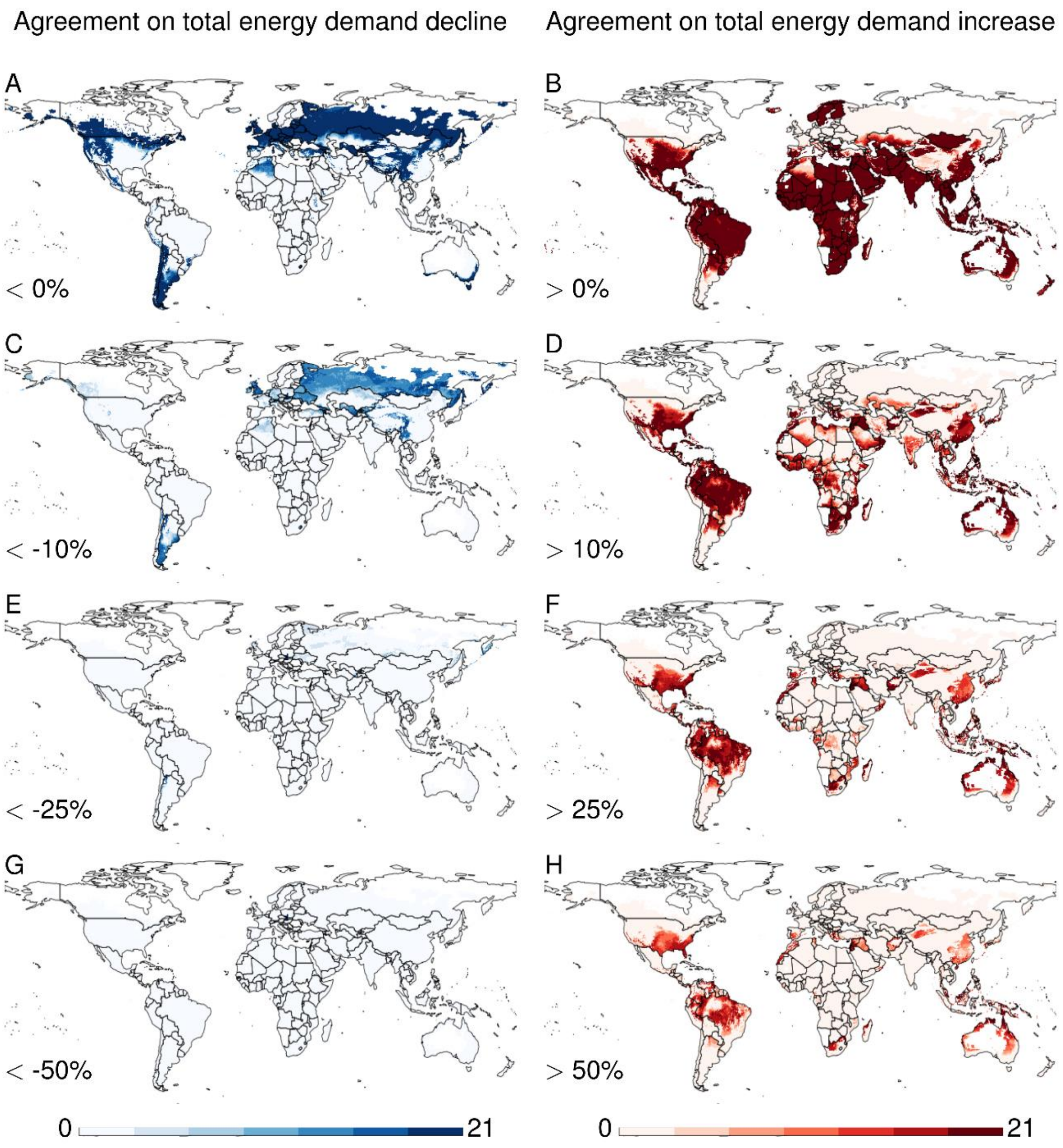

Supplementary Figure 10

Comparison of impacts on US electric power systems projected using econometric estimates from Auffhammer et al (2017) and De Cian and Sue Wing (2019), CCSM4 climate model.

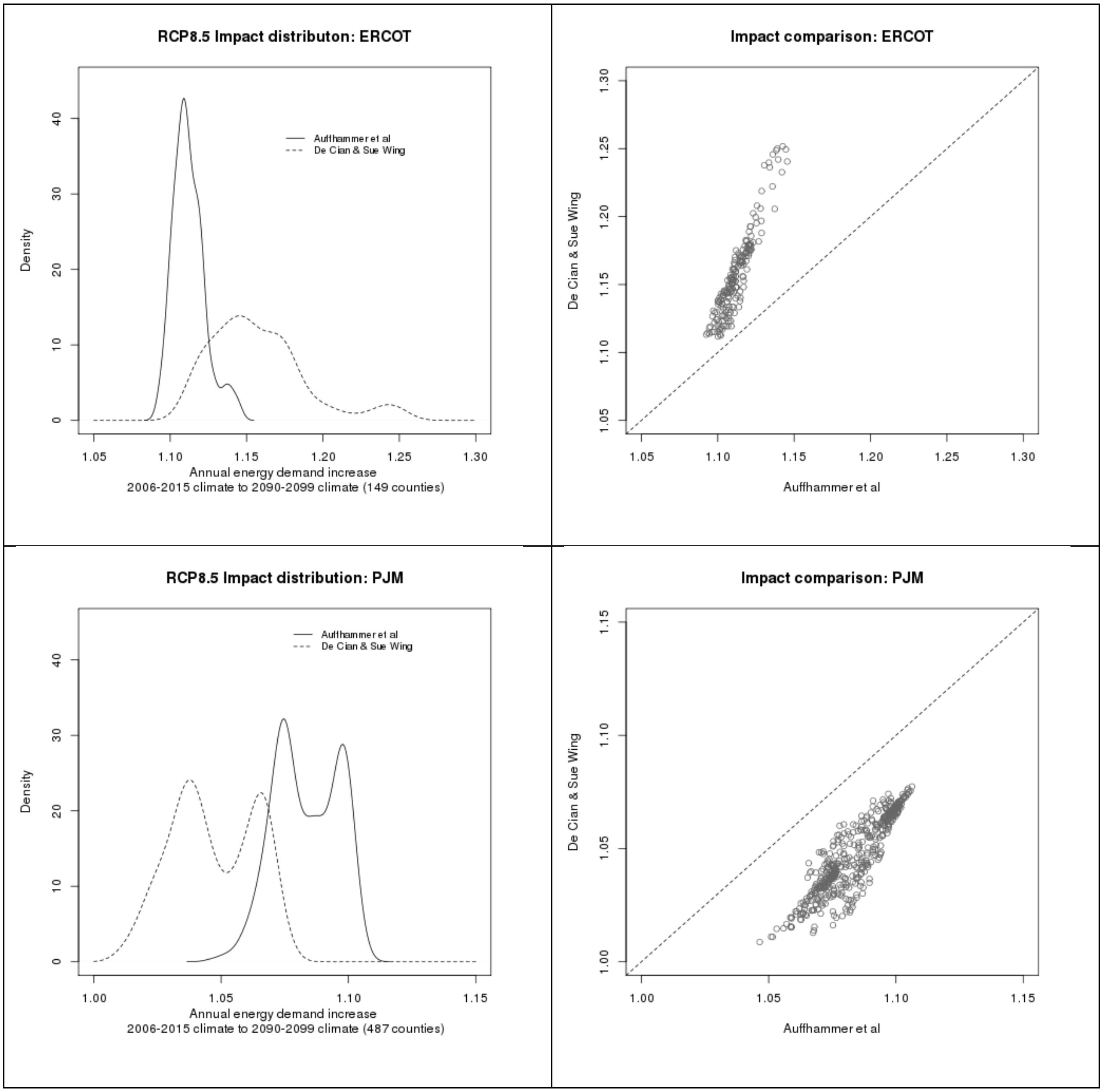

Supplementary Table 1

Absolute change in climate-related final energy demand (EJ/yr) by 2050 for all SSPs (part A) and additional change in energy demand due to climate change under RCP8.5 (part B) and RCP4.5 (part C). Parts B and C show the median and interquartile range across all 21 ESMs.

	SSP1	SSP2	SSP3	SSP4	SSP5
**A. Change in energy demand by 2050 from 2010 (EJ)**					
**Europe**	33	25	13	26	52
**North America**	31	26	16	28	49
**Oceania**	2	2	1	2	3
**South America**	9	7	7	6	11
**Middle East & Africa**	24	22	20	21	33
**Asia**	78	57	40	53	103
**World**	178	139	96	135	251
**B. Additional change in energy demand due to climate change (EJ/yr) under RCP8.5**					
**Europe**	-0.9	-0.1	0.6	-0.4	-2.4
	[-3.2,2.9]	[-2.2,4.3]	[-1.1,5.3]	[-2.4,4.5]	[-5.4,0.8]
**North America**	42	39	32	39	52
	[34.6,54.2]	[31.9,49.8]	[26.6,41.3]	[32.5,50.9]	[42.6,67.3]
**Oceania**	1.1	1.0	0.8	1.0	1.4
	[0.7,1.6]	[0.7,1.4]	[0.5,1.1]	[0.7,1.5]	[0.9,2]
**South America**	5.5	5.5	5.7	5.2	5.8
	[3.8,8.3]	[3.9,8.3]	[4.2,8.7]	[3.6,7.9]	[3.9,8.7]
**Middle East & Africa**	15.3	14.9	13.7	14.5	18.0
	[11.7,23.2]	[11.5,22.3]	[10.7,20.3]	[11.2,21.2]	[13.6,27.2]
**Asia**	60	52	44	50	70
	[34,87]	[31,75]	[27,65]	[29,73]	[39,101]
**World**	114	104	91	102	132
	[84,170]	[80,155]	[70,136]	[78,153]	[98,198]
**C. Additional change in energy demand due to climate change (EJ/yr) under RCP4.5**					
**Europe**	-3.0	-2.0	-1.3	-2.2	-4.9
	[-4,-2.5]	[-3,-1.5]	[-2.1,-0.8]	[-3.3,-1.7]	[-6.2,-4]
**North America**	21	19	16	19	25
	[14.6,30]	[13.5,27.7]	[11.2,22.9]	[13.6,28.1]	[17.8,37]
**Oceania**	0.3	0.3	0.2	0.3	0.4
	[0.2,0.5]	[0.2,0.5]	[0.2,0.4]	[0.2,0.5]	[0.3,0.6]
**South America**	2.4	2.5	2.7	2.3	2.5
	[2.2,3.7]	[2.2,3.8]	[2.4,4]	[2.1,3.5]	[2.3,3.9]
**Middle East & Africa**	8.5	8.3	7.7	8.1	9.8

	[5.7,9.4]	[5.6,9.2]	[5.2,8.6]	[5.5,9.1]	[6.6,11.1]
**Asia**	30	26	23	25	34
	[18,43]	[17,38]	[15,32]	[16,37]	[20,50]
**World**	60	55	49	54	69
	[38,80]	[36,74]	[33,65]	[35,72]	[43,93]

Supplementary Table 2

Percentage change in final energy demand as result of climate change by 2050 for all SSPs disaggregated for changes in hot and cold days under RCP8.5. Note that each median/interquartile range describes a different distribution of the 21 ESMs (for hot days, cold days and total) and therefore these summarized changes in hot/cold days do not add up to the total impacts. For each individual ESM realization, changes from hot and cold days do add up to the total impacts, though.

Region	Impact	SSP1	SSP2	SSP3	SSP4	SSP5
**Europe**	Total	-1% [-5%,4%]	0% [-4%,7%]	1% [-2%,11%]	-1% [-4%,7%]	-3% [-6%,1%]
	Hot days	11% [6%,23%]	11% [7%,25%]	12% [7%,27%]	12% [7%,26%]	10% [6%,22%]
	Cold days	-13% [-17%,-12%]	-12% [-16%,-12%]	-11% [-15%,-11%]	-13% [-17%,-12%]	-14% [-19%,-13%]
**North America**	Total	64% [53%,82%]	64% [53%,82%]	63% [52%,81%]	63% [52%,82%]	63% [51%,80%]
	Hot days	70% [58%,89%]	70% [58%,88%]	69% [58%,87%]	70% [58%,88%]	69% [57%,87%]
	Cold days	-6% [-7%,-5%]	-6% [-7%,-5%]	-6% [-7%,-5%]	-6% [-7%,-6%]	-7% [-8%,-6%]
**Oceania**	Total	28% [19%,41%]	28% [19%,41%]	29% [19%,41%]	28% [19%,41%]	28% [18%,41%]
	Hot days	32% [22%,44%]	32% [22%,44%]	32% [21%,44%]	32% [22%,45%]	32% [22%,45%]
	Cold days	-4% [-4%,-3%]	-3% [-3%,-3%]	-3% [-3%,-3%]	-3% [-3%,-3%]	-4% [-4%,-4%]
**South America**	Total	33% [23%,50%]	36% [25%,55%]	39% [29%,60%]	37% [26%,56%]	30% [20%,46%]
	Hot days	37% [27%,54%]	40% [30%,58%]	42% [32%,63%]	41% [30%,60%]	35% [26%,51%]
	Cold days	-4% [-5%,-4%]	-4% [-4%,-3%]	-3% [-3%,-3%]	-4% [-4%,-3%]	-5% [-5%,-4%]
**Middle East & Africa**	Total	37% [29%,57%]	39% [30%,58%]	38% [30%,56%]	39% [30%,57%]	37% [28%,55%]
	Hot days	40% [31%,60%]	41% [32%,61%]	40% [32%,59%]	41% [32%,60%]	39% [30%,58%]
	Cold days	-2% [-3%,-2%]	-2% [-3%,-2%]	-2% [-2%,-2%]	-2% [-3%,-2%]	-2% [-3%,-2%]
**Asia**	Total	50% [28%,72%]	52% [31%,76%]	54% [33%,79%]	52% [31%,77%]	48% [27%,70%]
	Hot days	55% [34%,80%]	57% [35%,83%]	58% [37%,84%]	58% [36%,84%]	54% [33%,78%]
	Cold days	-7% [-8%,-6%]	-6% [-6%,-5%]	-5% [-5%,-4%]	-6% [-7%,-6%]	-7% [-8%,-6%]
**World**	Total	36% [27%,54%]	37% [29%,56%]	39% [30%,58%]	37% [28%,56%]	34% [25%,51%]
	Hot days	44% [32%,61%]	44% [34%,62%]	45% [35%,64%]	44% [34%,63%]	42% [31%,59%]
	Cold days	-7% [-9%,-6%]	-7% [-8%,-6%]	-6% [-7%,-5%]	-7% [-8%,-6%]	-8% [-10%,-7%]

Supplementary Table 3

Percentage change in final energy demand as result of climate change by 2050 for all SSPs disaggregated for changes in hot and cold days under RCP4.5. Note that each median/interquartile range describes a different distribution of the 21 ESMs (for hot days, cold days and total) and therefore these summarized changes in hot/cold days do not add up to the total impacts. For each individual ESM, changes from hot and cold days do add up to the total impacts, though.

Region	Impact	SSP1	SSP2	SSP3	SSP4	SSP5
**Europe**	Total	-4% [-6%,-4%]	-3% [-5%,-3%]	-3% [-4%,-2%]	-4% [-5%,-3%]	-6% [-7%,-5%]
	Hot days	4% [3%,7%]	4% [3%,7%]	4% [3%,8%]	4% [3%,7%]	4% [2%,6%]
	Cold days	-10% [-12%,-7%]	-9% [-11%,-7%]	-9% [-10%,-6%]	-10% [-11%,-7%]	-11% [-12%,-8%]
**North America**	Total	31% [22%,46%]	31% [22%,46%]	31% [22%,45%]	31% [22%,45%]	30% [21%,44%]
	Hot days	37% [27%,51%]	37% [27%,51%]	36% [26%,50%]	36% [26%,51%]	36% [26%,50%]
	Cold days	-5% [-6%,-4%]	-4% [-5%,-4%]	-4% [-5%,-4%]	-5% [-6%,-4%]	-5% [-6%,-5%]
**Oceania**	Total	8% [6%,13%]	9% [6%,13%]	9% [7%,14%]	8% [6%,13%]	8% [5%,13%]
	Hot days	11% [9%,16%]	11% [9%,16%]	11% [9%,16%]	11% [9%,16%]	11% [9%,16%]
	Cold days	-3% [-3%,-3%]	-2% [-3%,-2%]	-2% [-2%,-2%]	-3% [-3%,-3%]	-3% [-4%,-3%]
**South America**	Total	15% [13%,23%]	16% [15%,25%]	19% [16%,28%]	17% [15%,25%]	13% [12%,20%]
	Hot days	18% [16%,26%]	20% [17%,28%]	21% [18%,30%]	20% [17%,28%]	17% [15%,24%]
	Cold days	-3% [-4%,-2%]	-3% [-3%,-2%]	-2% [-3%,-2%]	-3% [-3%,-2%]	-4% [-4%,-3%]
**Middle East & Africa**	Total	21% [14%,23%]	22% [15%,24%]	21% [15%,24%]	22% [15%,24%]	20% [13%,22%]
	Hot days	23% [16%,25%]	23% [16%,26%]	23% [16%,25%]	23% [16%,26%]	22% [15%,24%]
	Cold days	-2% [-2%,-1%]	-2% [-2%,-1%]	-2% [-2%,-1%]	-2% [-2%,-1%]	-2% [-2%,-1%]
**Asia**	Total	25% [15%,36%]	26% [17%,38%]	28% [18%,39%]	26% [17%,39%]	23% [14%,34%]
	Hot days	30% [20%,42%]	31% [21%,43%]	32% [22%,43%]	32% [21%,44%]	29% [19%,41%]
	Cold days	-5% [-6%,-4%]	-4% [-5%,-4%]	-4% [-4%,-3%]	-5% [-6%,-4%]	-5% [-7%,-5%]
**World**	Total	19% [12%,25%]	20% [13%,27%]	21% [14%,28%]	20% [13%,27%]	18% [11%,24%]
	Hot days	25% [17%,32%]	25% [17%,33%]	25% [18%,33%]	25% [18%,33%]	24% [16%,31%]
	Cold days	-6% [-7%,-4%]	-5% [-6%,-4%]	-4% [-5%,-3%]	-5% [-6%,-4%]	-6% [-7%,-5%]

Supplementary Table 4

Number of people (in millions) in developing regions exposed to increases in total energy demand of 25-50% and >50% under RCP8.5. Numbers indicate the median and interquartile ranges of the distribution over 21 ESMs. Countries are characterized by "adaptation challenge" based on current cutoff-levels of the World Bank for lower middle, upper middle and high income countries.

			SSP1	SSP2	SSP3	SSP4	SSP5
**South America**	High adaptation challenge	25-50%			1% [1%,2%]	1% [0%,2%]	
		>50%			1% [0%,1%]	1% [0%,2%]	
	Moderate adaptation chall.	25-50%	1% [1%,2%]	3% [3%,4%]	11% [10%,12%]	4% [3%,4%]	1% [0%,1%]
		>50%	1% [1%,1%]	2% [2%,4%]	19% [17%,25%]	3% [3%,5%]	0% [0%,1%]
	Low adaptation challenge	25-50%	13% [12%,14%]	11% [10%,12%]	2% [2%,3%]	10% [9%,10%]	13% [12%,14%]
		>50%	22% [18%,28%]	21% [18%,27%]	6% [5%,7%]	19% [17%,26%]	21% [17%,28%]
**Middle East & Africa**	High adaptation challenge	25-50%	0% [0%,0%]	6% [6%,6%]	16% [14%,18%]	16% [13%,19%]	0% [0%,0%]
		>50%	0% [0%,0%]	5% [3%,9%]	11% [8%,22%]	12% [9%,24%]	
	Moderate adaptation chall.	25-50%	15% [14%,19%]	13% [12%,14%]	4% [3%,5%]	3% [1%,4%]	6% [6%,8%]
		>50%	6% [4%,13%]	6% [3%,12%]	5% [3%,8%]	4% [3%,7%]	2% [1%,6%]
	Low adaptation challenge	25-50%	4% [3%,5%]	3% [2%,4%]	2% [2%,2%]	2% [2%,3%]	10% [9%,15%]
		>50%	6% [5%,9%]	5% [4%,7%]	3% [2%,4%]	4% [3%,5%]	9% [6%,13%]
**Asia**	High adaptation challenge	25-50%		0% [0%,0%]	1% [1%,2%]	1% [1%,2%]	
		>50%		1% [1%,1%]	1% [1%,2%]	1% [1%,2%]	
	Moderate adaptation chall.	25-50%	1% [1%,2%]	8% [6%,10%]	10% [8%,11%]	7% [5%,9%]	0% [0%,1%]
		>50%	3% [2%,4%]	4% [3%,6%]	8% [6%,10%]	4% [4%,6%]	1% [1%,1%]
	Low adaptation challenge	25-50%	12% [10%,14%]	5% [4%,13%]	4% [2%,10%]	5% [4%,13%]	12% [10%,16%]
		>50%	26% [15%,31%]	24% [14%,29%]	20% [11%,23%]	24% [14%,28%]	28% [15%,34%]

Supplementary Table 5

Share of population in developing regions exposed to increases in total energy demand of 25-50% and larger than 50%. Numbers indicate the median and interquartile range of the distribution over 21 ESMs. Countries are characterized by “adaptation challenge” based on present-day cutoff-levels of World Bank definition for lower middle, upper middle and high income countries.

			SSP1	SSP2	SSP3	SSP4	SSP5
**South America**	High adaptation challenge	25-50%			8 [4,10]	8 [3,10]	
		>50%			5 [1,10]	4 [1,9]	
	Moderate adaptation chall.	25-50%	7 [4,9]	20 [18,23]	75 [71,81]	22 [19,25]	6 [2,8]
		>50%	4 [3,6]	14 [10,24]	131 [113,168]	19 [15,28]	2 [0,3]
	Low adaptation challenge	25-50%	72 [65,75]	66 [58,69]	14 [13,17]	55 [50,58]	68 [62,74]
		>50%	117 [97,153]	125 [108,161]	39 [31,50]	112 [98,146]	111 [90,148]
**Middle East & Africa**	High adaptation challenge	25-50%	2 [1,2]	147 [139,157]	452 [391,512]	446 [353,511]	1 [1,2]
		>50%	0 [0,0]	117 [86,211]	326 [243,633]	341 [261,660]	
	Moderate adaptation chall.	25-50%	327 [306,419]	323 [302,343]	126 [93,148]	71 [31,100]	139 [134,177]
		>50%	129 [89,283]	142 [67,310]	132 [88,225]	123 [75,181]	51 [31,122]
	Low adaptation challenge	25-50%	92 [71,102]	71 [59,87]	56 [48,66]	67 [54,76]	209 [187,335]
		>50%	134 [101,201]	133 [101,174]	81 [62,119]	97 [72,135]	193 [133,277]
**Asia**	High adaptation challenge	25-50%		7 [5,10]	67 [54,109]	61 [49,92]	
		>50%		38 [30,41]	66 [47,80]	61 [45,75]	
	Moderate adaptation chall.	25-50%	56 [48,90]	368 [279,483]	487 [405,546]	291 [239,393]	17 [13,23]
		>50%	127 [81,154]	190 [160,279]	402 [325,522]	193 [174,275]	31 [26,36]
	Low adaptation challenge	25-50%	501 [428,596]	243 [169,589]	196 [113,526]	230 [157,561]	502 [433,671]
		>50%	1114 [652,1348]	1123 [666,1324]	1040 [557,1192]	1062 [621,1238]	1172 [641,1429]

Supplementary Table 6

Temperature and load control points, and degree day demand responses from ABH Fig. 1

	°C				MW				MW/°C			
	T_L	T_C	T_W	T_H	V_L	V_C	V_W	V_H	δ_L	δ_C	δ_W	δ_H
ERCOT	-1	12.5	21	27.5	13000	1000	5000	18000	489	400	1667	333
PJM	-8	12.5	21	27.5	25000	0	9000	33000	1220	0	3000	692

Supplementary Table 7

Comparison between DCSW elasticities and those estimated following equations S3 and S4.

(1) Sector	(2) De Cian & Sue Wing (2019) static estimates for temperate climate		(3) Reconciliation				(4) EIA SEDS Share of Electricity Sales	
	$\beta_{s,L}$	$\beta_{s,H}$	$\bar{\beta}_L$	$\bar{\beta}_H$	$\underline{\beta}_L$	$\underline{\beta}_H$	PJM	ERCOT
Residential	n.d.	0.010251‡					0.28	0.37
Commercial	0.0004681	0.0102889					0.57	0.34
Industrial	n.d.	0.003327†					0.13	0.28
Weighted Average	0.000167	0.008349	0.002016*	0.002696*	0.002017*	0.002724*		

n.d. Covariate dropped from regression,
* Significant at the 5% level, † Significant at the 10% level, ‡ Significant at the 15% level
